# Sebaceous Cysts—Conium

**Published:** 1883-07

**Authors:** Wm. Jefferson Guernsey

**Affiliations:** Frankford, Philadelphia


### SEBACEOUS CYSTS—CONIUM.

WM. JEFFERSON GUERNSEY, M. D., FRANKFORD.

The benign nature of the encysted, follicular, or sebaceous tumor, commonly called wen, has rendered it an unpopular subject for study, and caused dermatologists to summarily dismiss the thought of any other remedy than the usual surgical treatment. Very many patients, however, have a wholesome dread of anything in the line of an operation, and here, as in many another "surgical" affection, the homœopathic remedy should be substituted for the knife.

A sebaceous cyst is nothing more than an enormously distended sebaceous gland and duct, the walls of which are usually thickened and hypertrophied, even fibrinous or calcified, so as to form a tough sac or cyst. These cyst walls may, however, be thin and delicate. The inner layer is usually thin, as the corium is not implicated in its formation.

The contents are ordinarily firmly encysted and may be soft and cheezy, often semi-fluid or hard and friable. The mass is commonly composed of sebum, epidermic cells, crystals of cholesterin, and globules and crystals of fat. The contents may be yellow or white. In the large tumors it is frequently whitish, gray, and oily, while the smaller ones sometimes present concentric layers of epithelial cells and crystals of cholesterine (cholesteatoma). Those of some age are hard, dry, and laminated, and of blood corpuscles and pigment even to a brown, green or blackish hue.

In inflammation of the follicle, a greasy fluid is formed, mingled with pus and of a bad odor. The microscope has discovered rudimentary hairs also in the contents of these sacs.

---

*We feel justified in saying that this now famous resolution refers "solely to the consultation question"—*for its author says it does* and he ought to know. If this be its meaning, it is couched in such beautifully uncertain language that even a Talleyrand might envy its ambiguousness.

The skin covering sebaceous cysts is either normal in color or pale from extreme distension. They may be flattened and extended laterally or semiglobular or round, projecting prominently beyond the level of the skin, and as they increase in size showing a tendency to become pedunculated. They are quite variable as to size, and while writers generally compare their limits to that of a pea and walnut, they are to be found both smaller and larger. They occur singly or in great numbers, in children as well as adults; are unattended by pain. Their course is slow and they not unfrequently exist for years, or even a lifetime, without occasioning inconvenience. They may be hard to the feel, although generally soft and doughy. At times, when greatly distended, they break down and ulcerate, although they rarely do so from spontaneous inflammation. As a rule, they are freely movable. It has been stated, however, that when occurring upon the scalp they may largely develop, "induce inflammatory action in the pericranium, causing adhesive and cartilaginous degeneration of that portion lying next to the sac, and in rare cases absorption of the outer table of the skull result, forming a cup-shaped depression with slightly elevated edges." These humors are found chiefly upon the head, neck, forehead, face, eyelids, and more rarely the body. It should be noted that when occurring upon the scalp that portion is often devoid of hair. Dr. Helmuth relates an interesting case of a boy of eighteen months, from whom he removed a sebaceous cyst from the prepuce containing "a substance resembling cottage cheese in texture and swelling exactly like the secretion from the glands of Tyson."

Sebaceous cysts frequently appear to be hereditary, and are, no doubt, dependent upon a strumous diathesis. They should not be confounded with molluscum contagiosum. Molluscum was once described as existing in two forms: first, a hypertrophic growth of fibro-cellular tissue, called Molluscum fibrosum or Fibroma molluscum, but now better known as Fibroma; and second, the affection just spoken of, Molluscum contagiosum, so ably described by the late Tilbury Fox. The differential diagnosis of this affection from the one under consideration is easy. The contagious (so-called) enlargement of sebaceous glands can be readily recognized as "an umbilicated semi-transparent tumor, with a central opening from which sebaceous matter may be squeezed."

Now, what of treatment? Excision, unless the entire cyst be removed, is but temporary in relief, for the tumor will soon be reproduced.

Many other devices are used, but all savoring too strongly of surgery, which, as before intimated, is often objectionable, nay, should always be so when the desired object can be attained by a homœopathic remedy, for that medicine which will remove any result of morbid action in the system, however small that result or disease, will benefit the general health of the patient. The only remedies a long search has discovered are as follow:

In General: *Am. cb.*, Apis, Arsen., *Bary. cb.*, Bellad., Brom., Calc. c., Calc. flu., Caust., Clemat., Daph., Graph., Guar. t., Hell. ni., Hepar, Iodium, K. bic., K. brom., K. carb., K. iod., Lycopod., Merc. v., *Nit. ac.*, Nux v., Petrol., *Phos.*, *Ph. ac.*, Phytol., *Plumb.*, Pulsat., *Rhus*, Ruta, Sepia, *Silicea*, Sulph., Thuja, Zinc.

Head: Calc. c., Daph., Graph., K. cb., Hepar, Sil., Sulph.

Dunham recommended Calc. cb. for "encysted tumors of head and neck with fluid or semi-fluid contents."

All that Allen's *Register* shows is:

Under Sebaceous glands, affections of: K. br.

— — — inflammation of: Mor. sul.

— Scalp, tumors, old, become enlarged and sore when touched: Bary. c.

— — Wens, sensation as if pressed together by a ring or thimble: Glon.

— — Tumor: Bell.

— — — like blind boils: Fago.

— — — hard, subcutaneous: *Bell.*

— — — painless, suppurating: Phytol.

— — — stitches, worse at night: Gos.

Many of these symptoms are so vague as to be almost useless, yet they are suggestive.

I have had little experience with any of the remedies just mentioned. A few years ago, while treating a patient with *Conium*, I found a large wen of the scalp much reduced in size, and, though opposed to reliance upon clinically obtained symptoms, I could not refrain from further experiment with this medicine. I have since used it in so many cases of wen in persons suffering from no other complaint that unless contra-indicated I think of it at once. Let me, in self-defense, call attention to the fact that wens are almost invariably free from any subjective symptoms, so that the "indications" for a remedy, unless of a general nature, are absent.

Two months ago a gentleman requested the removal of a wen upon the posterior portion of the thigh, exactly in that locality upon which the edge of a chair seat came in contact while sitting. It was about the size of an English walnut, and from its location caused much inconvenience. I gave him Conium, and in two weeks the tumor was less than three-fourths its original size. He called again in about a week to ask if he had not better discontinue the *medicine* (*Sac. lac.*), as the tumor could not be found, nor could he designate the exact spot where it had been. My manner of giving the remedy has been a powder every night for two weeks. No doubt three or four doses would answer as well, but I have never tried it. I use invariably Fincke's 70m. Once I tried the 30th of Bœricke, but without the least change, while, in the same patient, after waiting a period without medicine, Con. 70m entirely removed the tumor in a few weeks.

I desire to further state that this same medicine and potency has *never* failed me in the removal of milium of the eyelids, for which I use it in the same manner unless contra-indicated.

When we can, let us not disturb nature with the knife or other rash treatment, but aid her to assume a healthy condition and rid herself of these petty defects by a close adherence to our faith.

